# Exploring the impact of pandemic fear on visitation to park attractions in urban city: A case study in Seoul, South Korea

**DOI:** 10.1371/journal.pone.0301869

**Published:** 2024-04-16

**Authors:** Yunwon Choi

**Affiliations:** 1 Climate & Environment Data Center, Gyeonggi Research Institute, Jangan-gu, Suwon City, Gyeonggi Province, Republic of Korea; 2 Research Institute of Agriculture and Life Sciences, Seoul National University, Seoul, Republic of Korea; Tokyo Medical and Dental University: Tokyo Ika Shika Daigaku, JAPAN

## Abstract

This research explores changes in perceptions and utilization of parks during the COVID-19 pandemic in Seoul, South Korea. It investigates the relationship between fear of the pandemic and individuals’ opinions about open spaces and their visiting decisions. The study surveyed 600 adults from February 22–23, 2022, and used structural equation modeling to analyze the data. The findings revealed that increased fear of the pandemic led to more positive park sentiments, resulting in higher park visits and fewer visits to other public spaces. The research highlights the significance of parks during the COVID-19 pandemic and how people’s perceptions were influenced by their pandemic-related fear.

## 1. Introduction

The COVID-19 pandemic was initially detected in 2019 and spread across continents in 2020. As of October 2023, more than 697 million cases, along with approximately 6.9 million deaths, had been reported [1]. The pandemic has presented a range of health and medical challenges, including preventing virus transmission, which placed immense strain on healthcare systems and hospitals [2]. COVID-19 also gave rise to other problems, such as social isolation, mental health concerns, economic hardships, educational gaps, and the disruption of communities and societies. Research suggests that these phenomena arose owing to the implementation of social distancing measures to curb the spread of the virus in numerous countries [3].

Fear of COVID-19 and its mental health consequences were significant and far-reaching. Increased stress was caused by numerous factors, including fear of virus transmission, social isolation, the uncertain duration of the pandemic, family conflicts, and financial difficulties [4]. Some media outlets have reported that the psychological distress experienced during the pandemic may be even more severe than the physical symptoms caused by COVID-19 infection. When severe, these challenges can become potential precursors that contribute to suicidal behavior and domestic violence within families [4–7].

Depression and anxiety also showed a staggering 25% increase in global prevalence in the first year of the pandemic [8]. While multiple stress factors have been identified, this increase could be mainly explained by stay-at-home measures based on pandemic fears [9], which contributed to heightened feelings of loneliness, subsequently leading to increased rates of depression and anxiety [10]. Combined with a lack of activity and sunlight, the decrease in outdoor physical activity significantly contributed to an increase in mental health problems among individuals [11–14].

These phenomena highlight the devastating consequential impact of COVID-19 on mental health, emphasizing the need for policy initiatives, medical interventions, and urban planning support to address these effects for all age groups.

### 1.1 Use of urban parks during the pandemic

Urban Parks and green spaces have contributed to reducing the psychological suffering brought about by COVID-19 [15]. While the pandemic led to a decline in outings owing to social norms and fear of infection, visits to green spaces surged by over 50% worldwide (Google, 2022). Visitors to urban parks increased in most countries from February 16, 2020, compared to pre-COVID-19 pandemic levels [16,17].

Seoul, South Korea also exhibited this trend, as of October 15, 2022, with a 47% increase in the number of park visitors compared to the pre-pandemic period [17]. Additionally, there has been a rapid increase in the percentage of people who prefer landscaped and vacant spaces as leisure areas in apartment complexes since the onset of the pandemic [18].

The growing demand for parks and outdoor green spaces during the pandemic highlights the important advantages offered by urban parks [19]. To alleviate heightened stress, fear and anxiety, many residents actively sought nearby locations as an alternative to indoor activities that posed a higher risk of virus transmission [20,21], opting instead to visit urban parks as alternative venues for recreation and relaxation [22]. Urban parks are safe and preferred destinations for leisure and relaxation, thereby reducing fear levels and making them ideal sanctuaries for individuals seeking solace.

The Centers for Disease Control and Prevention of the U.S. [23] encouraged physical activities in parks, trails, and open spaces to help individuals alleviate fear and stress, enjoy fresh air, and sustain an active lifestyle during the pandemic [24]. Parks, thus, served as sanctuaries, offering people opportunities for physical activity and mental relaxation [25]. Recent reports suggest that people recognized green spaces as nature-friendly areas with low transmission risk [26], which could have positively impacted how they responded to the effects of the COVID-19 pandemic. Studies have found an association between increased leisure time spent in green spaces during the pandemic and residents’ expressing improved happiness [27]. For example, a Canadian study found that among adults who were not physically active, there was a positive relationship between engaging in more outdoor activities during the pandemic and higher levels of well-being [28]. However, reports from several cities throughout the pandemic have highlighted the lack of equity in parks, leading many to recognize inequalities in park access and subsequent physical and mental health disparities during pandemic, even within the same city [29,30]. Hence, to prepare for future pandemics, it is essential to examine the role of parks, the motivations for park visits during a pandemic, and explore equitable park planning to ensure this function can be carried out throughout the city.

Additionally, people who experience high levels of stress in their daily lives, whether there is a pandemic or not, may benefit from visiting parks and spending time in open spaces, which can offer opportunities for social interaction and reconnection with nature [31]. Spending time in nature, including parks and green spaces, can positively affect mental health and well-being and can lower levels of the stress hormone cortisol [32]. Engaging in physical activities like walking, jogging, cycling, or simply breathing fresh air can alleviate stress, anxiety, and depressive symptoms while promoting relaxation and rejuvenation [33,34].

he benefits of these parks should be maximized in future pandemic scenarios, as preventive medicine and epidemiological studies have also indicated that the emergence of new COVID-19 variants remains a possibility, and similar epidemics may occur due to environmental degradation and climate change [35,36]. Consequently, it is imperative to develop planning for parks and open spaces that citizens can utilize for their health in the event of future pandemics. Prior to this, it is essential to investigate the factors that influenced people’s use of parks during the COVID-19 pandemic, including their emotional state before and after park visits, changes in their perceptions of parks, and how park usage differed based on their residential environment. However, research on these factors is currently lacking.

In this backdrop, this study investigates changes in the perceptions of parks, utilization of public spaces, and actual park visits in Seoul in relation to individuals’ fear of COVID-19 during the pandemic. Specifically, I aimed to address the following research question: Do fears of the COVID-19 pandemic influence individuals’ opinions about open spaces and their decisions to visit them? The study employed structural equation modeling (SEM) to examine the causal relationships among the variables of fear of the pandemic (FEAR), outings to public spaces during the pandemic (OUTING), opinions on open spaces/parks during the pandemic (PARK), and visitation to open spaces/parks during the pandemic (VISIT). I hypothesized:

**Hypothesis 1**: The more people fear the pandemic, the more positive feelings they have toward parks.
**Hypothesis 2**: The more people fear the pandemic, the more they visit parks.
**Hypothesis 3**: The more people fear the pandemic, the fewer public activities, other than visiting open spaces, they engage in during the pandemic.

## 2. Materials and methods

### 2.1 Study site: Seoul, South Korea

#### 2.1.1 Open spaces of Seoul

**Fig 1:**
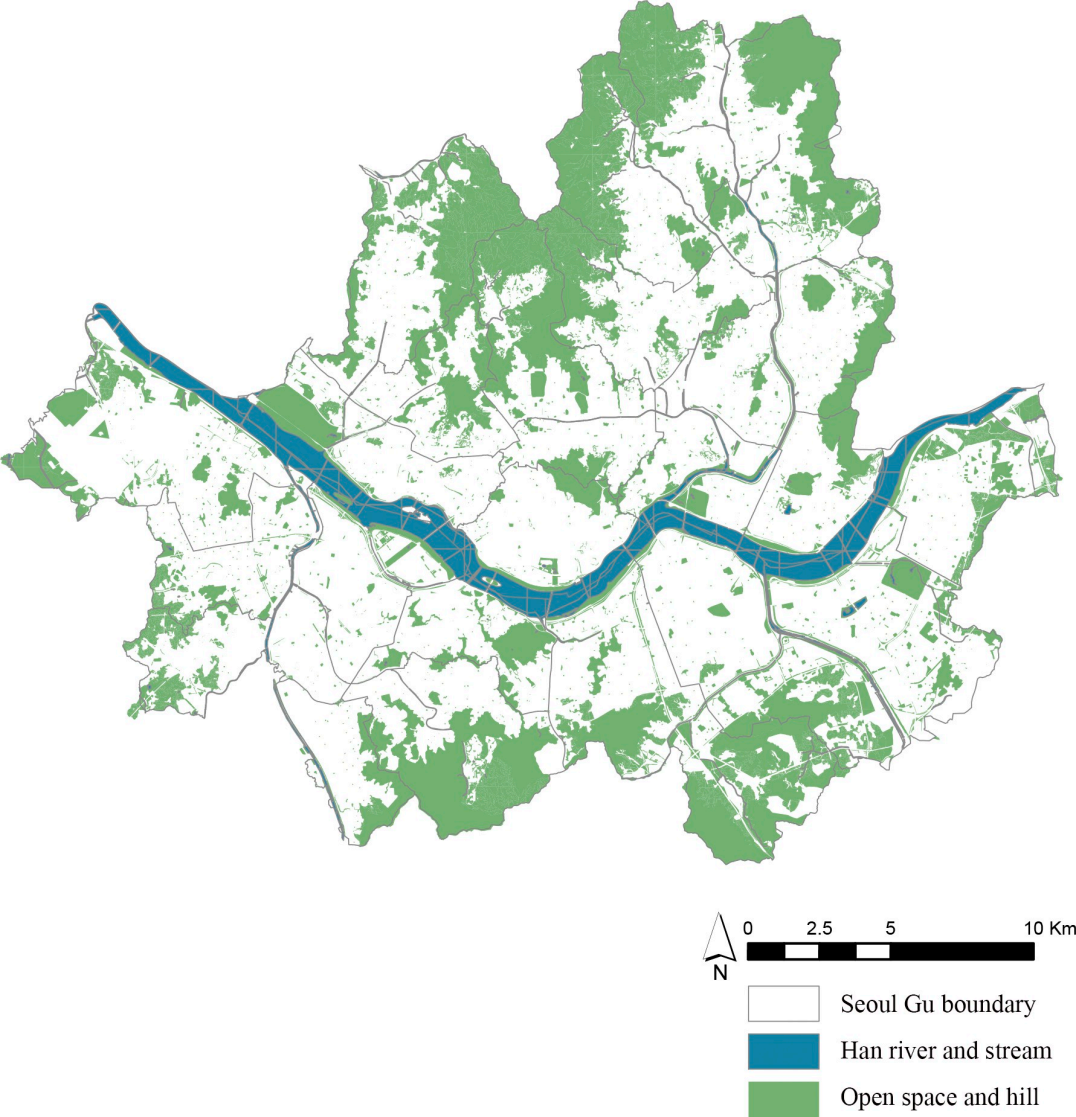
Study site—Seoul.

This study was conducted in Seoul (Fig 1), a metropolitan city and the capital of South Korea. As of 2020, Seoul had the highest population density in South Korea, with more than 15,800 people residing per square kilometer [37].

The total area of urban forested areas in Seoul, which includes all natural and manmade green areas, expanded from 168.16 km^2^ in 2009 to 172.68 km^2^ in 2022. This increase can be credited to both land restoration initiatives and the expansion of urban forested areas within residential zones, which include diverse green spaces like small parks, roadside greenery, school forests, and others. These developments ensure convenient access from residential areas. These areas increased from 31.1 km^2^ in 2009 to 47.3 km^2^ in 2021.These developments increased Seoul’s park coverage to 28.53%, with 2,959 locations in 2022, providing an urban park area per capita of 17.74 m^2^). However, Seoul’s natural environment is primarily composed of forests at its boundary, and the portion of parks accessible on foot within the city remains limited at just 5.65 m^2^ per person [38].

However, this number of Seoul falls short of these global standards. Recently, the United Nations introduced a green space guideline of 30 m^2^ per person [39], while the European Union has set its standard at 26 m^2^ per person [40]. In the United States, the standard is 18 m^2^ per person, as determined by the Public Health Service and the Department of Housing [39], while the WHO recommends a minimum of 9 m^2^ per person [41,42]. Therefore, Seoul has a pressing need to create more parks and open spaces in the city, given the possibility of recurring pandemics.

In addition to the provision of open spaces, accessibility is another crucial aspect. Ongoing analysis and proposals focus on ensuring access to green spaces within walking distance [43]. The European Environment Agency suggests a maximum distance of approximately 1,000 meters (equivalent to a 15-minute walk), while English Nature recommends an even shorter distance of up to 300 meters [44].

To enhance park accessibility, linear parks have been introduced among various types of parks and open spaces [45,46]. Linear parks offer access from various areas and establish natural connections with the surrounding environment, providing diverse and enduring experiences. Extensive research has explored the cultural, social, economic, and health benefits associated with linear park designs [47]. One notable example is New York City’s world-famous High Line, which has significantly piqued public interest in these spaces [48].

Similarly, in Seoul, the Gyeongui Line Forest Park, repurposed from an abandoned railroad, garnered significant attention and underwent revitalization as a linear park [49]. This successful transformation has inspired more local governments in Korea, including Seoul, to explore the development of linear parks [50]. Furthermore, experts argue that linear parks are ideal for the post-COVID-19 era, as they provide linear spaces for physical activities like walking, enabling people to maintain social distancing. This contrasts with traditional parks, which often encourage closer interactions and gatherings [51,52].

#### 2.1.2 COVID-19 regulations in Seoul

In the context of Seoul’s response to the pandemic, it is important to note that despite the current high overall number of confirmed COVID-19 cases, the city effectively controlled the pandemic by employing the 3T (Trace-Test-Treat) strategy until February 2022, which marked the midpoint of the pandemic [53].

Seoul achieved commendable results despite its high population density, primarily through enhanced contact tracing and the management of confirmed cases [54,55]. Like other Korean cities, Seoul’s strategy involved identifying and closely monitoring individuals who tested positive for COVID-19 [56]. Epidemiological investigators, the Seoul Infectious Disease Control Support Group, and public health centers collaborated to trace confirmed cases using mobile phone data, credit card records, and details of public transportation usage [57]. Information about confirmed cases was updated daily on the Seoul Metropolitan Government website, and citizens were kept informed through disaster alert texts and other communication channels [58].

Wearing a mask and checking temperature when entering indoor facilities were mandatory throughout the pandemic period, becoming cultural norms [59]. The government implemented social distancing and quarantine guidelines in stages, adjusting them in response to the number of confirmed cases. Unlike some countries, however, the Korean government avoided implementing a full lockdown that restricted all outings and gatherings [60]. Instead, regulations were primarily imposed on infected individuals, who were prohibited from leaving their homes for two weeks. Public places, such as restaurants and markets, visited by infected individuals were temporarily closed. High-risk facilities like sports events and entertainment venues had visitor regulations. Schools conducted remote classes, while organizations and companies actively encouraged remote work and flexible hours, although this wasn’t mandatory for private companies [61].

In this context, though people had the freedom to decide whether to go out, many refrained from doing so owing to the prevailing social atmosphere and fear of infection. However, as the pandemic progressed, spaces such as parks became more popular because of their lower risk of infection and the opportunity they provided for maintaining physical and mental health through physical activity [62].

### 2.2 Survey data and sample

I conducted a survey using the Opensurvey mobile application from February 22 to 23, 2022, with 600 adult men and women residing in Seoul, South Korea. The participants were randomly selected from the Opensurvey panels based on sex and age compositional proportions. The survey comprised 69 questionnaires. Participants gave their consent before completing the survey by clicking “Yes” after being presented with an informative text. This study was approved by Institutional Review Board (IRB) at Seoul National University (IRB number: 2202/003-008).

### 2.3 Statistical analysis: Structural equation modeling

**Fig 2:**
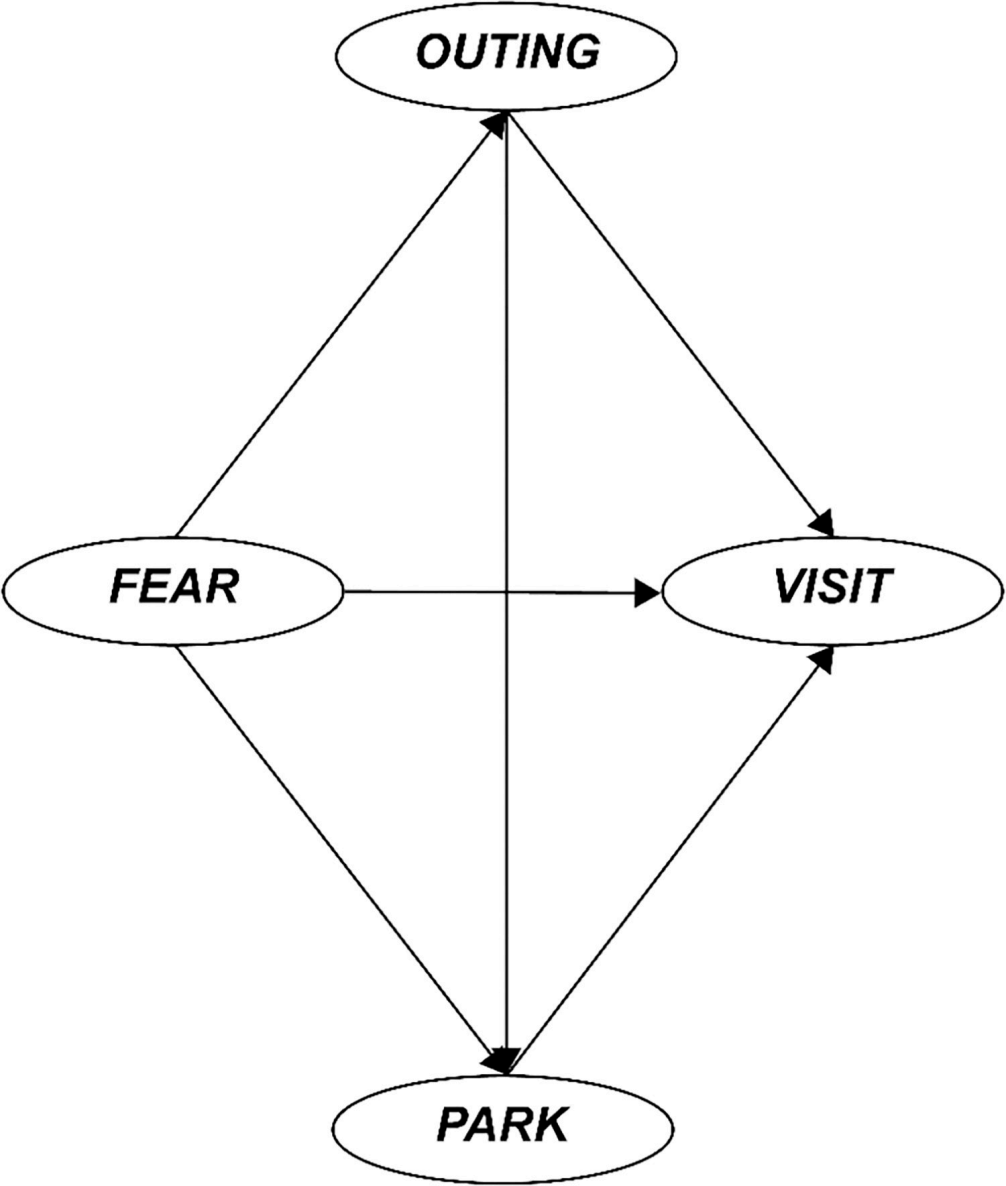
Research model.

Structural equation modeling (SEM) was utilized to investigate the relationships among four latent variables: FEAR, OUTING, PARK, and VISIT (Fig 2). Unlike traditional regression models, SEM can simultaneously test multiple hypotheses, investigate direct and indirect effects, define causal relationships, and evaluate measurement errors. Furthermore, the structural error in SEM, which represents the unexplained portion of the variance of the dependent variable in a regular regression model, is explicitly specified and controlled within the SEM framework. This ensures that the parameter estimates remain unbiased. SEM provides a comprehensive, flexible approach for analyzing complex relationships between variables, making it valuable for validation or exploratory purposes [63,64].

In SEM, latent variables are used to represent abstract concepts or constructs that cannot be directly measured, such as psychological traits, attitudes, or behaviors. SEM allows these latent variables to be inferred or estimated from multiple observed variables. As such, it is a powerful tool for understanding and visualizing complex relationships in data and is used in various fields, particularly in conjunction with the social sciences and psychology. I constructed the SEM model using analytical results from several previous studies that influenced our hypotheses [65,66].

SPSS Amos 29.0 was used for model analysis. Amos is employed across various fields, including urban planning [67].

### 2.4 Variables: Constructs and indicators

Our analysis comprised four constructs: FEAR, OUTING, PARK, and VISIT. VISIT was the dependent variable. These four constructs represent abstract concepts that require observed variables to be estimated.

#### 2.4.1 FEAR: Fear of COVID-19

Fear can motivate people to take precautions and protect themselves from COVID-19. Governments and authorities implemented various regulations and recommendations to mitigate the spread of the virus, including travel restrictions, stay-at-home orders, strict handwashing, and mask mandates [68]. Implementing social distancing measures has been observed to reduce the spread of the virus and its associated impacts. There is a relationship between fear of a pandemic and the extent to which people abided by such protective measures [65]. Individuals with more fear of COVID-19 were more inclined to adopt preventive behaviors than people who had lower levels of fear [66].

The Fear of COVID-19 Scale (FCV-19S) was developed in 2020 to measure public concern about COVID-19. The questionnaire includes questions such as, “Do you worry about catching COVID-19?” and “Are you scared of COVID-19?” [69]. However, given that participants’ responses and behaviors toward COVID-19 are influenced not only by fear but also by cultural, governmental, and resource-related factors, alternative scales have been developed [70]. Accordingly, this study evaluated fear of COVID-19 by assessing the degree to which individuals complied with recommended protective measures, considering Korea’s pre-COVID-19 hygiene rules and response to the pandemic [68]. To construct the latent variable FEAR and measure the extent of fear about COVID-19, this study included a questionnaire that assessed the degree of compliance with various COVID-19 safety rules in Korea. The measured variables for the latent variable FEAR comprised handwashing; frequent ventilation; following coughing etiquette; avoiding touching the ears, nose, and throat with dirty hands; maintaining social distance; avoiding crowded places; and not sharing food.

Compliance with COVID-19 recommendations is determined by various factors, including fear of the disease, susceptibility to disinformation, and belief in the effectiveness of interventions [65,71]. However, political beliefs and moral values may also play a role in compliance with preventive measures [72,73]. Unlike behavioral recommendations, hesitancy or refusal to receive a COVID-19 vaccine may be influenced by various trust-related factors and may not necessarily represent a lack of fear of the virus. Some individuals may be hesitant to get vaccinated owing to safety and efficacy concerns or believe misinformation or conspiracy theories about vaccines [74], while others may have religious or philosophical objections. There may also be a lack of trust in the healthcare system or government. Access barriers can also be reasons for vaccine hesitancy. Therefore, this study did not adopt people’s responses to whether they had been vaccinated as a measurement variable.

#### 2.4.2 OUTINGS: Outings during the COVID-19 pandemic

During the pandemic, almost 90% of respondents canceled all plans for outings or travel for 2020 [75]. Although conditions such as lockdowns also affected fear of COVID-19, individual fear levels had a greater impact. Individuals with low fear levels were less affected by going out, whereas those with high fear levels reported difficulty leaving home [76]. People were even afraid to leave their homes to buy basic daily necessities [77]. The number of outings, including visits to restaurants, cafes, and pubs, fluctuated but was consistently impacted throughout the pandemic [78].

This study established a relationship between fear and outings. Three measurement variables were used to measure the latent variable OUTING, reflecting time spent on nonessential activity in indoor public spaces, contravening the COVID-19 guidelines. Eating out, using cafes, and using shopping malls were included when constructing the latent variable OUTING. Visiting religious spaces was not taken into consideration, as survey respondents did not share the same religious status. Additionally, going to the park was excluded because it was a dependent variable in this study and occurs outdoors.

#### 2.4.3. PARK: Perceptions of open spaces/parks during the COVID-19 pandemic

Spending time in nature can have positive effects on mental health [20,79]. During the pandemic, individuals sought ways to alleviate stress and anxiety. The need for parks increased since people were searching for outdoor areas in which to engage in physical activities and socialize while adhering to social distancing guidelines. The accessibility and availability of parks during the pandemic differed based on the level of restrictions in place and the location [80]. This increased demand underscores the significance of public green spaces for physical and mental well-being.

The latent variable PARK indicated changes in perceptions of open spaces and parks as people experienced the COVID-19 pandemic. The PARK construct comprised six variables that gauged respondents’ views on the importance of parks in general, safety from COVID-19 in parks, the significance of parks as spaces during a pandemic, the need for more parks after a pandemic, the fear of contracting COVID-19 while visiting parks, whether stress was alleviated after a park visit, and whether respondents wanted to visit parks more frequently after their initial visit.

#### 2.4.4. VISIT: Visitation to open spaces and parks during the COVID-19 pandemic

During the pandemic, park visits increased globally, while the number of visitors to other public spaces either showed less significant increases or experienced decreases [17]. However, the factors that influenced people to choose parks over other locations remain unclear. This study assumed that fear of the pandemic motivated individuals to seek outdoor spaces such as parks.

VISIT was constructed with two measured variables obtained from the survey.

## 3. Results

### 3.1. Sample characteristics

The survey results were not separately analyzed for male and female groups. However, the gender ratio of the survey participants was designed by considering the male-to-female ratio of adults in Seoul, which was approximately 47.3:52.6 in 2022 [37]. Among the 600 observations in the sample, the survey employed a 50:50 gender ratio by rounding the numbers. Table 1 shows socioeconomic characteristics of the survey respondents.

**Table 1:** Socioeconomic characteristic of the survey respondents.

Variable	Level	Frequency	Percent
Gender	Male	300	50
	Female	300	50
Age	20s	120	20
	30s	120	20
	40s	120	20
	50s	120	20
	60s+	120	20
Marital status	Single	348	58.0
	Married	220	36.7
	Other (Divorced, separated, etc.)	32	5.3
Educational level	Middle school graduate or below	6	1.0
	High school graduate	125	20.8
	Bachelor	392	65.3
	Post-Graduate degree	77	12.8
Total Household income (monthly)	Less than 1 million won	16	2.7
	1–2 million won	47	7.8
	2–3 million won	100	16.7
	3–5 million won	171	28.5
	5–7 million won	121	20.2
	more than 7 million won	145	24.2
Household size	1 person	99	16.5
	2 people	130	21.7
	3 people	170	28.3
	4 people	168	28.0
	**5 people and more**	**33**	**5.5**

### 3.2. Estimating measurement models

Indicators that displayed multicollinearity, had low internal consistency reliability, or did not show statistical significance were excluded when integrating the four latent constructs—FEAR, OUTING, PARK, and VISIT. Table 2 shows the factor loading of the indicators, as well as the average variance extracted (AVE) and Cronbach’s alpha values for each construct. All constructs demonstrated satisfactory Cronbach’s alpha and AVE values.

**Table 2:** Measurement information and reliability checks.

Indicator	Factor Loading	AVE	Construct Reliability (α)
**FEAR**		0.421	0.834
Washing hands	0.605 ***		
Ventilation	0.628 ***		
Coughing etiquette	0.479 ***		
Avoiding touching one’s ears, nose, and mouth	0.728 ***		
Social distancing	0.723 ***		
No populated space	0.690 ***		
No sharing of food	0.656 ***		
**OUTING**		0.523	0.765
Eating out	0.735 ***		
Café	0.801 ***		
Indoor mall	0.622 ***		
**PARK**		0.480	0.844
Parks are important regardless of COVID-19	0.292***		
Parks offer a safe space from COVID-19	0.354 ***		
Parks are more important in the post-COVID era	0.337 ***		
Experience: Stress relief	0.942 ***		
Wanted to visit parks more often after using them	0.942 ***		
Felt healthier after using parks	0.910 ***		
**VISIT**		0.465	0.629
Frequency of visitation	0.558***		
Frequency of visits to the nearest park	0.787 ***		

** p < 0.05, *** p < 0.01.

I evaluated the model fit for our structure, which incorporated the four individual constructs, by examining various fit indices: χ2(CMIN/df) = 3.973, GFI (goodness of fit) = 0.860, CFI (comparative fit index) = 0.863, RMSEA (root mean square error of approximation) = 0.050, TLI (Tucker–Lewis Index) = 0.839 (Table 3). The covariance between the measurement error terms of the indicators following the upper limit of the correction index was set at 4.00, and all were statistically significant at the 5% level.

**Table 3:** Model fit.

χ2	GFI	CFI	RMSEA	TLI
(CMIN/df)	(Goodness of Fit)	(Comparative Fit Index)	(Root Mean Square Error of Approximation)	(Tucker–Lewis Index)
3.973	0.86	0.863	0.05	0.839

### 3.3. Analytical results

**Fig 3:**
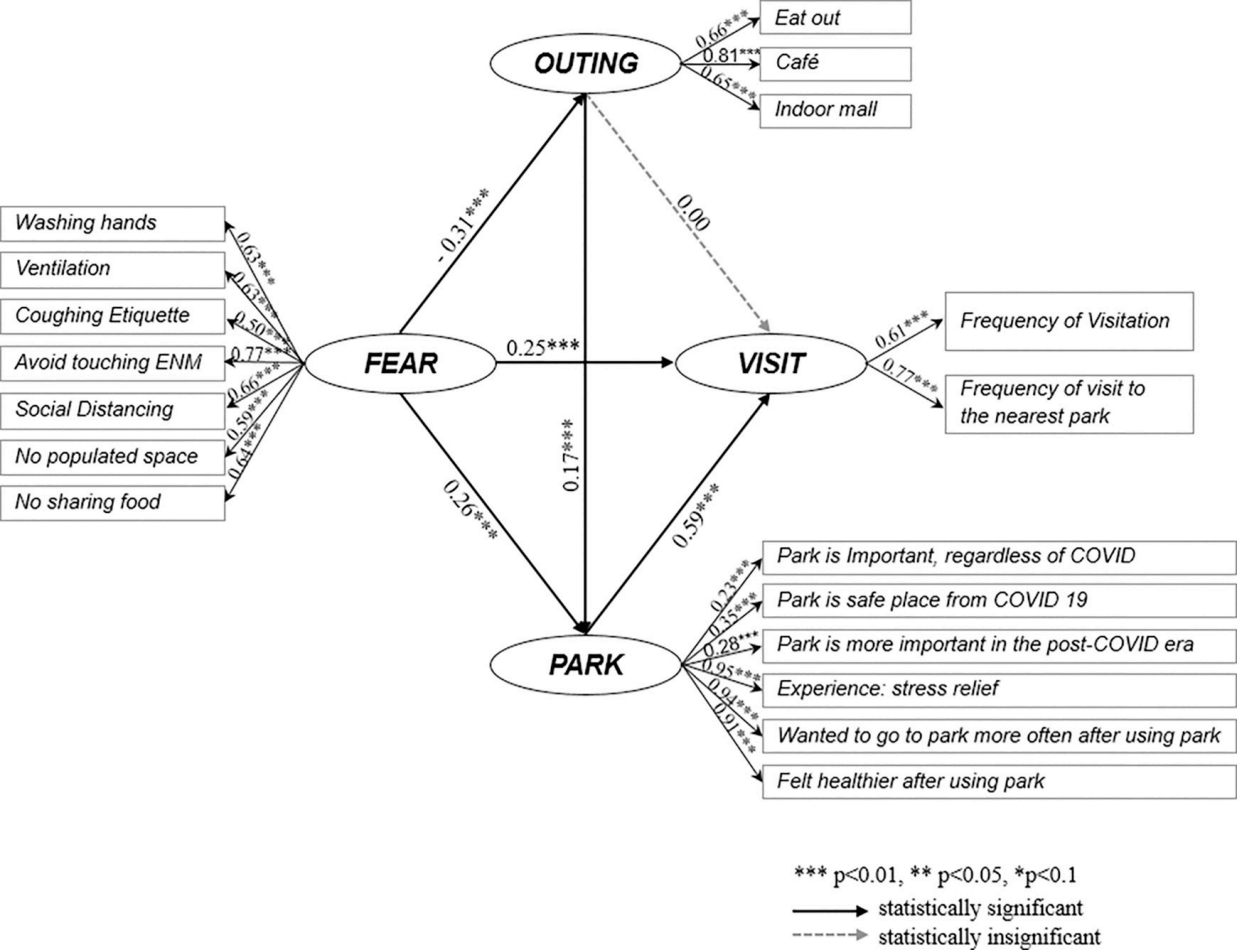
SEM results.

I present the SEM results in Fig 3, which depicts hypotheses 1, 2, and 3. The results indicate that, at a statistical significance level of 5%, FEAR positively influenced participants’ perceptions of using parks and park visits during the pandemic but negatively affected their engagement in activities in other public places.

In the SEM analysis, hypothesis 1, "The more people fear the pandemic, the more positive feelings they have toward parks," was supported as FEAR was positively correlated with PARK (coeff. = 0.26), indicating that as participants’ fear of COVID-19 increased, their perceptions of using parks during the pandemic became more favorable. Out of the six indicators of FEAR, avoiding touching one’s ears, nose, and mouth with dirty hands had the greatest impact on pandemic fears (coeff. = 0.77). Social distancing had the second most significant impact (coeff. = 0.66), while refraining from sharing food was the third most influential factor (coeff. = 0.64). The experience of stress relief from using parks during the pandemic had the greatest influence on constructing the PARK index (coeff. = 0.95). Additionally, individuals’ perceptions of parks were positively influenced by the realization of the value of using parks more frequently after their initial visits (coeff. = 0.94) and feeling healthier after visiting a park (coeff. = 0.91). Individuals who utilized parks at least once during the pandemic reported positive experiences and maintained significantly positive perceptions of the value and role of parks during this period.

The findings supported hypothesis 2, "The more people fear the pandemic, the more they visit parks," as well. FEAR was positively correlated with VISIT (coeff. = 0.25), indicating that as fear of the pandemic increased, participants were more likely to visit parks. Among the two indicators of VISIT, the frequency of visits to parks nearer residential areas (coeff. = 0.61) was higher than the overall frequency of park visits (coeff. = 0.77).

Hypothesis 3, "The more people fear the pandemic, the fewer public activities, other than visiting open spaces, they engage in during the pandemic," was also supported by analytical results. The impact of FEAR on OUTING was negative (coefficient = -0.38), suggesting that as fear of the pandemic increased, participants’ inclination to visit public places other than parks decreased. Hypothesis 3 was thus supported. This phenomenon may be attributed to the heightened concern that there was a greater risk of infection in these public spaces. Among the three indicators of OUTING, "going to a café" exhibited the highest factor value (coeff. = 0.81). It can be inferred that going to a café was considered an optional activity during the pandemic when compared to dining out or visiting indoor shopping malls.

## 4. Discussion

Park visitors significantly increased during the COVID-19 pandemic [81,82]; however, analyses of the factors driving this trend are lacking. This study examined the impact of fear of the pandemic on participants’ perceptions of parks, park visits, and utilization of other public spaces. The analysis showed a causal relationship between fear of the pandemic, participants’ inclination to visit parks, and their positive perceptions of them. The results confirmed all three hypotheses within the context of Seoul: As fear of the pandemic increased, participants’ positive feelings toward parks grew, resulting in more park visits and fewer outings to other public spaces.

The finding that people had a more positive view of parks when they feared the pandemic more may be due to the belief that parks present a lower infection risk than other outdoor city. Additionally, there has been increased interest in nature since the pandemic, possibly influenced by the positive emotional connection to parks and green spaces [83]. Fresh air, breezes, water, trees, flowers, insects, animals, and soothing sounds contribute to the mental well-being provided by parks as they cannot be replicated within indoor spaces. These circumstances likely influenced the change in people’s perceptions of parks as their fear of the pandemic increased.

To assess fear of the pandemic, the study analyzed individuals’ adherence to COVID-19 hygiene guidelines. Before the pandemic, Korea did not have established hygiene norms to prevent infection and virus transmission [68]; therefore, adherence to sanitation standards, which people have newly recognized as important, is an indicator of the level of fear. The most significant aspect of fear was people’s avoidance of touching their ears, noses, and mouths with unwashed hands. Social isolation also demonstrated significant factor value, likely motivated by fear or a heightened awareness of its importance in a densely populated city like Seoul. The practice of refraining from sharing food, which contradicts Korean cuisine’s style on group dining [84], was the third most significant factor. The actions people took during the pandemic in defiance of these cultural norms show their efforts to stop the spread of the virus and overcome their fear of COVID-19. The finding that increased park visitation corresponded to greater fear of the pandemic aligns with the observed rise in park visits during the pandemic [17]. What distinguishes this analysis is that it establishes a causal relationship between actively seeking out parks and the intensification of fears about the pandemic.

Furthermore, people minimized unnecessary trips to public areas (other than parks) where crowds gather owing to the potential for spreading the infection. Access to indoor facilities in Korea was heavily regulated during the pandemic, with only those with a normal body temperature being allowed entry. At one point, a vaccine pass and personal information were required for access to indoor facilities, restaurants, and cafés [85]. These measures were implemented to create a safe environment in these indoor spaces, leading people to naturally avoid public venues (other than parks) as their concerns about the pandemic grew stronger.

Parks have long been recognized for providing various benefits to both humans and ecosystems [86]. During the pandemic, they served as spiritual sanctuaries, offering a sense of safety amid fear [25]. However, as mentioned earlier, inequality in park access has impacted leisure opportunities and hindered the fair utilization of city parks as a spatial resource to respond to the pandemic. Ultimately, this affects people’s emotion and their physical and mental health. As parks have a significant positive impact on people’s lives, both during and outside of a pandemic, ensuring park equity is crucial [87,88]. Governments should address social inequality in parks to ensure equitable access and supply by providing parks of different sizes, types, and programs in easily accessible locations.

This study emphasizes the importance of theoretical and practical knowledge about the value of parks, as well as the lessons learned from the COVID-19 crisis. However, it has some limitations. First, the study period only covered February 2022, excluding the entire COVID-19 pandemic period. Given the multiple waves of the pandemic, people’s fear of it and their behaviors might have varied. For a more comprehensive evaluation, this study should be supplemented with more surveys or a panel survey that covers a range of time periods. Second, the study focused on Seoul, and the responses and behaviors of individuals in other global cities or in smaller towns and rural areas within South Korea could differ. Expanding the scope of the study to include various locations could, thus, provide additional insights.

## 5. Conclusion

Urban parks and open spaces offer spaces where city residents can lead physically and mentally healthy and active lives. They also play an important role as sanctuaries that promote overall physical and mental well-being during a pandemic. This study provides analytical findings on the importance of parks and adds to the growing evidence regarding the correlation between people’s fear of the pandemic and their attitudes and utilization of parks during the COVID-19 pandemic. Pandemic-related research and the experiences gained from the COVID-19 pandemic will help prepare for the many recreational and mental health challenges that a new pandemic may bring.

To maximize the advantages of urban parks, whether during a pandemic or not, governments should develop strategies to optimize the use of park facilities, considering size, number, distribution, programs, and management methods to ensure easy access for the larger population. In numerous countries, the provision of parks in cities is significantly influenced by tax revenues, leading to disparities in park access particularly impacting health inequalities during pandemics. Nonetheless, local authorities must remain committed to providing parks and open spaces in an equitable manner, ensuring they are easily accessible to all citizens, not only in normal times but also during special situations like a pandemic, where they can serve as sanctuaries for alleviating fear and promoting mental and physical health. Thus, research on budget-friendly park planning or parks that are easily accessible to a larger population is crucial.

This study provides a foundation for future research. For example, one approach is dividing groups based on their responses to questions about the park environment around their residences. This would allow for an analysis of whether people’s perceptions and use of parks during the pandemic differed based on park equity. Additionally, analytical studies using big data can explore the relationship between factors like the daily number of COVID-19 cases and the size, shape, and programming of parks that were popular during the pandemic.

The study’s findings also have implications for urban design and planning, benefiting urban designers, planners, and policymakers in developing pandemic-resilient landscape guidelines. Implementing these findings in cities can promote citizens’ physical and mental well-being both during pandemics and in their everyday lives.

## Supporting information

S1 File (XLSX)

S1 Appendix (DOCX)

## References

[1] WorldOMeter. Coronavirus toll update: Cases & deaths by country [Internet]. Worldometer. 2023 [cited 2023 Nov 19]. Available from: https://www.worldometers.info/coronavirus/.

[2] Tuttle KR. Impact of the COVID-19 pandemic on clinical research. Nature Reviews. Nephrology. 2020; 16: 562–564. doi: 10.1038/s41581-020-00336-9 32760016 PMC7404075

[3] O’Regan D, Jackson ML, Young AH, Rosenzweig I. Understanding the impact of the COVID-19 pandemic, lockdowns and social isolation on sleep quality. Nature and Science of Sleep. 2021;13:2053–2064. doi: 10.2147/NSS.S266240 34795545 PMC8593898

[4] Sher L. The impact of the COVID-19 pandemic on suicide rates. QJM: Monthly Journal of the Association of Physicians. 2020;113:707–712. doi: 10.1093/qjmed/hcaa202 32539153 PMC7313777

[5] Lim LJH, Fong LM, Hariram J, Lee YW, Tor PC. COVID-19, a pandemic that affects more than just physical health: Two case reports. Asian Journal of Psychiatry. 2020;53:102200. doi: 10.1016/j.ajp.2020.102200 32540751 PMC7282747

[6] Brailovskaia J, Teismann T, Friedrich S, Schneider S, Margraf J. Suicide ideation during the COVID-19 outbreak in German university students: comparison with pre-COVID 19 rates. Journal of Affective Disorders Reports. 2021;6:00228. doi: 10.1016/j.jadr.2021.100228 34632441 PMC8488981

[7] Tanaka T, Okamoto S. Increase in suicide following an initial decline during the COVID-19 pandemic in Japan. Nature Human Behaviour. 2021;5:229–238. doi: 10.1038/s41562-020-01042-z 33452498

[8] World Health Organization. COVID-19 pandemic triggers 25% increase in prevalence of anxiety and depression worldwide [Internet]. World Health Organization. 2022 [cited 2023 Nov 19]. Available from: https://www.who.int/news/item/02-03-2022-covid-19-pandemic-triggers-25-increase-in-prevalence-of-anxiety-and-depression-worldwide.

[9] Kauhanen L, Wan Mohd Yunus WMA, Lempinen L, Peltonen K, Gyllenberg D, Mishina K, Gilbert S, Bastola K, Brown JSL, Sourander A. A systematic review of the mental health changes of children and young people before and during the COVID-19 pandemic. European Child and Adolescent Psychiatry. 2023;32:95–1013. 10.1007/s00787-022-02060-0 PMC9373888 35962147

[10] Hoffart A, Johnson SU, Ebrahimi OV. Loneliness during the COVID-19 pandemic: change and predictors of change from strict to discontinued social distancing protocols. Anxiety, Stress, and Coping. 2022;35:44–57. doi: 10.1080/10615806.2021.1958790 34314285

[11] Choi Y, Yoon H, Kim D. Where do people spend their leisure time on dusty days? Application of spatiotemporal behavioral responses to particulate matter pollution. The Annals of Regional Science. 2019;63:317–339.

[12] Violant-Holz V, Gallego-Jiménez MG, González-González CS, Muñoz-Violant S, Rodríguez MJ, Sansano-Nadal O, Guerra-Balic M. Psychological health and physical activity levels during the COVID-19 pandemic: a systematic review. International Journal of Environmental Research and Public Health. 2020;17:9419. doi: 10.3390/ijerph17249419 33334073 PMC7765528

[13] Wolf S, Seiffer B, Zeibig JM, Welkerling J, Brokmeier L, Atrott B, Ehring T, Schuch FB. Is physical activity associated with less depression and anxiety during the COVID-19 pandemic? A rapid systematic review. Sports Medicine. 2021;51:1771–1783. doi: 10.1007/s40279-021-01468-z 33886101 PMC8060908

[14] Chen T, Lucock M. The mental health of university students during the COVID-19 pandemic: an online survey in the UK. PLOS ONE. 2022;17:e0262562. doi: 10.1371/journal.pone.0262562 35020758 PMC8754313

[15] Freeman S, Eykelbosh A. COVID-19 and outdoor safety: Considerations for use of outdoor recreational spaces. National Collaborating Centre for Environmental Health. 2020;829:1–15.

[16] Geng DC, Innes J, Wu W, Wang G. Impacts of COVID-19 pandemic on urban park visitation: a global analysis. Journal of Forestry Research. 2021;32:553–567. doi: 10.1007/s11676-020-01249-w 33204057 PMC7660132

[17] Google. See how your community moved differently due to COVID-19 [Internet]. COVID-19 Community Mobility Reports. 2022 October 17. [cited 2023 Nov 15]. Available from https://www.google.com/covid19/mobility/.

[18] Ministry of Culture, Sports and Tourism. National leisure life survey. Ministry of Culture, Sports and Tourism; 2023 Mar.

[19] Poortinga W, Bird N, Hallingberg B, Phillips R, Williams D. The role of perceived public and private green space in subjective health and wellbeing during and after the first peak of the COVID-19 outbreak. Landscape and Urban Planning. 2021;211:104092. doi: 10.1016/j.landurbplan.2021.104092 36540159 PMC9754643

[20] Venter ZS, Barton DN, Gundersen V, Figari H, Nowell M. Urban nature in a time of crisis: Recreational use of green space increases during the COVID-19 outbreak in Oslo, Norway. Environmental Research Letters. 2020;15:104075.

[21] Lu Y, Zhao J, Wu X, Lo SM. Escaping to nature during a pandemic: A natural experiment in Asian cities during the COVID-19 pandemic with big social media data. Science of the total environment. 2021;777:146092.

[22] Kleinschroth F, Kowarik I. COVID‐19 crisis demonstrates the urgent need for urban greenspaces. Frontiers in Ecology and the Environment. 2020;18:318–319. doi: 10.1002/fee.2230 32834788 PMC7436739

[23] Centers for Disease Control and Prevention. Visiting Parks and Recreational Facilities: Protect Yourself and Others from COVID-19 [Internet]. Centers for Disease Control and Prevention. 2020 June. [cited 2023 Nov 15]. Available from: https://stacks.cdc.gov/view/cdc/89194.

[24] Gubić I, Wolff M. Use and design of public green spaces in Serbian cities during the COVID-19 pandemic. Habitat International. 2022;128:102651. doi: 10.1016/j.habitatint.2022.102651 36061218 PMC9420699

[25] Alkhaja N, Alawadi K, Ibrahim HM. Post-pandemic planning: do we have enough and efficient access to parks? Frontiers in Built Environment. 2023;9:1158430.

[26] Lee W, Kim H, Choi HM, Heo S, Fong KC, Yang J, Park C, Kim H, Bell ML. Urban environments and COVID-19 in three Eastern states of the United States. Science of the Total Environment. 2021;779:146334. doi: 10.1016/j.scitotenv.2021.146334 33744577 PMC7952127

[27] Cheng Y, Zhang J, Wei W, Zhao B. Effects of urban parks on residents’ expressed happiness before and during the COVID-19 pandemic. Landscape and Urban Planning. 2021;212:104118. doi: 10.1016/j.landurbplan.2021.104118 36569996 PMC9757897

[28] Lesser IA, Nienhuis CP. The impact of COVID-19 on physical activity behavior and well-being of Canadians. International Journal of Environmental Research and Public Health. 2020;17:3899. doi: 10.3390/ijerph17113899 32486380 PMC7312579

[29] Pipitone JM, Jović S. Urban green equity and COVID-19: Effects on park use and sense of belonging in New York City. Urban Forestry and Urban Greening. 2021;65:127338.

[30] Lee S, Lee C, Xu M, Li W, Ory M. People living in disadvantaged areas faced greater challenges in staying active and using recreational facilities during the COVID-19 pandemic. Health and Place. 2022;75:102805. doi: 10.1016/j.healthplace.2022.102805 35443226 PMC9013405

[31] Bowler DE, Buyung-Ali LM, Knight TM, Pullin AS. A systematic review of evidence for the added benefits to health of exposure to natural environments. BMC Public Health. 2010;10:456. doi: 10.1186/1471-2458-10-456 20684754 PMC2924288

[32] Ward Thompson CW, Roe J, Aspinall P, Mitchell R, Clow A, Miller D. More green space is linked to less stress in deprived communities: evidence from salivary cortisol patterns. Landscape and Urban Planning. 2012;105:221–229.

[33] Wan C, Shen GQ. Encouraging the use of urban green space: the mediating role of attitude, perceived usefulness and perceived behavioural control. Habitat International. 2015;50:130–139.

[34] Mayen Huerta CM, Utomo A. Evaluating the association between urban green spaces and subjective well-being in Mexico City during the COVID-19 pandemic. Health and Place. 2021;70:102606. doi: 10.1016/j.healthplace.2021.102606 34139612 PMC9760010

[35] Carlson CJ, Albery GF, Merow C, Trisos CH, Zipfel CM, Eskew EA, Olival KJ, Ross N, Bansal S. Climate change increases cross-species viral transmission risk. Nature. 2022;607:555–562. doi: 10.1038/s41586-022-04788-w 35483403

[36] World Health Organization. Weekly epidemiological update on COVID-19 −20 July 2023. World Health Organization; 2023 Jul.

[37] Statistics Korea. Regional population and population density [Internet]. E-nara index. Statistics Korea; 2023 [cited 2023 Nov 19]. Available from: https://www.index.go.kr/unity/potal/main/EachDtlPageDetail.do?idx_cd=1041.

[38] Seoul Solution. Mayor Oh Se-hoon announces the “Garden City Seoul” plan, ensuring that Seoul turns green 365 days a year, wherever you go [Internet]. Seoul Solution. 2023 [cited 2023 Nov 19]. Available from: https://seoulsolution.kr/ko/content/9909.

[39] Laghai H. and Bahmanpour H. (2012), GIS Application in Urban Green space Per Capita Evaluation, Annals of Biological Research, 3(5), pp 2439–2446.

[40] Vlad M. I. and Brătăşanu D., (2011), Quality of life assessment based on spatial and temporal analysis of the vegetation area derived from satellite images, Romanian review of regional studies, 7(2), pp 111–120.

[41] Saz S. D. and Rausell P., (2008), A Double-Hurdle model of urban green areas valuation: Dealing with zero responses, Landscape and Urban Planning, 84, pp 241–251.

[42] Addas A., Maghrabi A. A proposed planning concept for public open space provision in Saudi Arabia: a study of three Saudi cities. International Journal of Environmental Research and Public Health. 2020;17(16):5970. doi: 10.3390/ijerph17165970 32824590 PMC7459717

[43] Moran MR, Rodríguez DA, Cotinez-O’Ryan A, Miranda JJ. Park use, perceived park proximity, and neighborhood characteristics: evidence from 11 cities in Latin America. Cities. 2020;105:102817. doi: 10.1016/j.cities.2020.102817 33012941 PMC7490577

[44] Khalil R. Quantitative evaluation of distribution and accessibility of urban green spaces (case study: City of Jeddah). International Journal of Geomatics and Geosciences. 2014;4(3):526–535.

[45] Brown G, Schebella MF, Weber D. Using participatory GIS to measure physical activity and urban park benefits. Landscape and Urban Planning. 2014;121:34–44.

[46] Molnar D. Anatomy of a Park. Illinois: Waveland Press; 2015.

[47] Gusteler F, López R, Faggi A. Models for a better management of linear parks. In: In 3rd Pan-American Interdisciplinary Conference, PIC 2017. 2017. p. 270.

[48] Yoon H. When and where do we see the proximity effect of a new park?–A case study of the Dream Forest in Seoul, Korea. Journal of Environmental Planning and Management. 2018;61:1113–1136.

[49] Kim SR, Choi Y, Yoon H. The analysis of the visitors’ experiences in Yeonnam-dong before and after the Gyeongui line park project-a text mining approach. Journal of the Korean Institute of Landscape Architecture. 2019;47(4):33–49.

[50] Jung E, Choi Y, Yoon H. The impact of the Gyeongui Line Park project on residential property values in Seoul, Korea. Habitat International. 2016;58:108–117.

[51] Elfakharany MM, Naguib IM, Abdelhamid MM Post-pandemic urban robustness and public health in congested cities. Engineering Research Journal. 2022;174:285–307.

[52] Sung H, Kim WR, Oh J, Lee S, Lee PSH. Are all urban parks robust to the COVID-19 pandemic? Focusing on type, functionality, and accessibility. International Journal of Environmental Research and Public Health. 2022;19(10):6062. doi: 10.3390/ijerph19106062 35627599 PMC9141827

[53] Anttiroiko AV. Successful government responses to the pandemic: contextualizing national and urban responses to the COVID-19 outbreak in east and west. International Journal of E-Planning Research. 2021;10(2):1–17.

[54] Park S, Choi GJ, Ko H. Information technology–based tracing strategy in response to COVID-19 in South Korea—privacy controversies. JAMA. 2020;323(21):2129–2130. doi: 10.1001/jama.2020.6602 32324202

[55] Huang JH, Floyd MF, Tateosian LG, Hipp JA. Exploring public values through Twitter data associated with urban parks pre-and post-COVID-19. Landscape and Urban Planning. 2022;227:104517. doi: 10.1016/j.landurbplan.2022.104517 35966883 PMC9358034

[56] Bode M, Craven M, Leopoldseder M, Rutten P, Wilson M. Contact tracing for COVID-19: New considerations for its practical application | McKinsey [Internet]. www.mckinsey.com. 2020 [cited 2023 Nov 19]. Available from: https://www.mckinsey.com/industries/public-sector/our-insights/contact-tracing-for-covid-19-new-considerations-for-its-practical-application.

[57] Lee D, Lee J. Testing on the move: South Korea’s rapid response to the COVID-19 pandemic. Transportation Research Interdisciplinary Perspectives. 2020;5:100111. doi: 10.1016/j.trip.2020.100111 34171015 PMC7172645

[58] Lee JY, Kim M, Jhon M, Kim JW, Ryu S, Kim JM, Kim SW. Factors associated with a negative emotional response to news media and nationwide emergency text alerts during the covid-19 outbreak in Korea. Psychiatry Investigation. 2021;18(9):825. doi: 10.30773/pi.2021.0087 34500508 PMC8473856

[59] Song S, Choi Y. Differences in the COVID-19 pandemic response between South Korea and the United States: a comparative analysis of culture and policies. Journal of Asian and African Studies. 2023;58(2):196–213. 38603407 10.1177/00219096221137655 PMC9669502

[60] Kim S, Kim YJ, Peck KR, Ko Y, Lee J, Jung E. Keeping low reproductive number despite the rebound population mobility in Korea, a country never under lockdown during the COVID-19 pandemic. International Journal of Environmental Research and Public Health. 2020;17(24):9551. doi: 10.3390/ijerph17249551 33419347 PMC7765860

[61] Korea Center for Disease Control and Prevention. All about Korea’s Response to COVID-19. Cheongju, Korea: Korea Center for Disease Control and Prevention; 2020.

[62] Kim J, Ko Y, Kim W, Kim G, Lee J, Eyman OTG, Chowdhury S, Adiwal J, Son Y, Lee WK. Understanding the impact of the COVID-19 pandemic on the perception and use of urban green spaces in Korea. International Journal of Environmental Research and Public Health. 2023;20(4):3018. doi: 10.3390/ijerph20043018 36833712 PMC9962542

[63] Ullman JB, Bentler PM. Structural equation modeling. in Handbook of Psychology, second ed. Vol. 2 Chapter 23; 2012.

[64] Choi Y, Yoon H. Do the walkability and urban leisure amenities of neighborhoods affect the body mass index of individuals? Based on a case study in Seoul, South Korea. International Journal of Environmental Research and Public Health. 2020;17:2060. doi: 10.3390/ijerph17062060 32244911 PMC7142730

[65] Jørgensen F, Bor A, Petersen MB. Compliance without fear: individual‐level protective behaviour during the first wave of the COVID‐19 pandemic. British Journal of Health Psychology. 2021;26:679–696. doi: 10.1111/bjhp.12519 33763971 PMC8250211

[66] Harper CA, Satchell LP, Fido D, Latzman RD. Functional fear predicts public health compliance in the COVID-19 pandemic. International Journal of Mental Health and Addiction. 2021;19:1875–1888. doi: 10.1007/s11469-020-00281-5 32346359 PMC7185265

[67] Hahm Y, Yoon H, Choi Y. The effect of built environments on the walking and shopping behaviors of pedestrians; a study with GPS experiment in Sinchon retail district in Seoul, South Korea. Cities. 2019;89:1–13.

[68] Choi K, Sim S, Choi J, Park C, Uhm Y, Lim E, Kim AY, Yoo SJ, Lee Y. Changes in handwashing and hygiene product usage patterns in Korea before and after the outbreak of COVID-19. Environmental Sciences Europe. 2021;33:79. doi: 10.1186/s12302-021-00517-8 34249592 PMC8254429

[69] Ahorsu DK, Lin CY, Imani V, Saffari M, Griffiths MD, Pakpour AH. The Fear of COVID-19 Scale: development and initial validation. International Journal of Mental Health and Addiction. 2022;20:1537–1545. 32226353 10.1007/s11469-020-00270-8 PMC7100496

[70] Håkansson A, Claesdotte E. Fear of COVID-19, compliance with recommendations against virus transmission, and attitudes towards vaccination in Sweden. Heliyon. 2022;8:e08699. doi: 10.1016/j.heliyon.2021.e08699 34981036 PMC8716143

[71] Roozenbeek J, Schneider CR, Dryhurst S, Kerr J, Freeman ALJ, Recchia G, van der Bles AM, Van Der Linden S. Susceptibility to misinformation about COVID-19 around the world. Royal Society Open Science. 2020;7:201199. doi: 10.1098/rsos.201199 33204475 PMC7657933

[72] Wang D, Marmo-Roman S, Krase K, Phanord L. Compliance with preventative measures during the COVID-19 pandemic in the USA and Canada: results from an online survey. Social Work in Health Care. 2021;60:240–255. doi: 10.1080/00981389.2020.1871157 33407057

[73] Díaz R, Cova F. Reactance, morality, and disgust: the relationship between affective dispositions and compliance with official health recommendations during the COVID-19 pandemic. Cognition and Emotion. 2022;36:120–136. doi: 10.1080/02699931.2021.1941783 34132171

[74] Troiano G, Nardi A. Vaccine hesitancy in the era of COVID-19. Public Health. 2021;194:245–251. doi: 10.1016/j.puhe.2021.02.025 33965796 PMC7931735

[75] Hussain MW, Mirza T, Hassan MM. Impact of COVID-19 pandemic on the human behavior. International Journal of Education and Management Engineering. 2020;10:35–61.

[76] Nishad MFR, Sarker PC, Sugawara D. Factors contributing to fear of COVID-19 and its consequence in mental health. International Journal of Indian Psychology. 2023;11:173–180.

[77] Singh J, Singh J. COVID-19 and its impact on society. Electronic Research Journal of Social Sciences and Humanities. 2020; 2.

[78] Smith LE, Potts HW, Amlȏt R, Fear NT, Michie S, Rubin GJ. Engagement with protective behaviours in the UK during the COVID-19 pandemic: a series of cross-sectional surveys (the COVID-19 rapid survey of adherence to interventions and responses [CORSAIR] study). BMC Public Health. 2022;22(1):1–11. 35272652 10.1186/s12889-022-12777-x PMC8907902

[79] Young DR, Hong BD, Lo T, Inzhakova G, Cohen DA, Sidell MA. The longitudinal associations of physical activity, time spent outdoors in nature and symptoms of depression and anxiety during COVID-19 quarantine and social distancing in the United States. Preventive Medicine. 2022;154:106863. doi: 10.1016/j.ypmed.2021.106863 34774881 PMC8717103

[80] Csomós G, Borza EM, Farkas JZ. Exploring park visitation trends during the Covid-19 pandemic in Hungary by using mobile device location data. Scientific Reports. 2023;13:11078. doi: 10.1038/s41598-023-38287-3 37422583 PMC10329667

[81] Roll U, Jarić I, Jepson P, da Costa‐Pinto AL, Pinheiro BR, Correia RA, Malhado AC, Ladle RJ. COVID‐19 lockdowns increase public interest in urban nature. Frontiers in Ecology and the Environment. 2021;19:320–322. doi: 10.1002/fee.2374 34518761 PMC8426885

[82] Isabella M, Claudia B, Giulia CM, Alessandro C, Alessandro P. Citizens’ use of public urban green spaces at the time of the COVID-19 pandemic in Italy. Urban Forestry and Urban Greening. 2022;77:127739. doi: 10.1016/j.ufug.2022.127739 36168321 PMC9499986

[83] Park S, Kim S, Lee J, Heo B. Evolving norms: social media data analysis on parks and greenspaces perception changes before and after the COVID 19 pandemic using a machine learning approach. Scientific Reports. 2022;12:13246. doi: 10.1038/s41598-022-17077-3 35918495 PMC9344807

[84] Shahidi F. Do traditional food cultures play a role in COVID-19 for health and immunity? Journal of Food Bioactives. 2021;14.

[85] Korea Disease Control and Prevention Agency. Are you curious about the vaccine pass? Q&A guide for step-by-step daily recovery-7 [Internet]. Korea Disease Control and Prevention Agency. 2021 [cited 2023 Nov 19]. Available from: https://www.kdca.go.kr/gallery.es?mid=a20503010000&bid=0002&b_list=9&act=view&list_no=145428&nPage=1&vlist_no_npage=1&keyField=&keyWord=&orderby=.

[86] Basu S, Nagendra H. Perceptions of park visitors on access to urban parks and benefits of green spaces. Urban Forestry and Urban Greening. 2021;57:126959.

[87] Jian IY, Chan EH, Xu Y, Owusu EK. Inclusive public open space for all: spatial justice with health considerations. Habitat International. 2021;118:102457.

[88] Bustamante G, Guzman V, Kobayashi LC, Finlay J. Mental health and well-being in times of COVID-19: a mixed-methods study of the role of neighborhood parks, outdoor spaces, and nature among US older adults. Health and Place. 2022;76:102813. doi: 10.1016/j.healthplace.2022.102813 35623164 PMC9127349

