# Supplementary material for: Exploring the impact of pandemic fear on visitation to park attractions in urban city: A case study in Seoul, South Korea
Source: PLoS One. 2024 Apr 16;19(4):e0301869. doi: 10.1371/journal.pone.0301869 (PMC11020851; doi:10.1371/journal.pone.0301869)
Supplement: S1 Appendix — (DOCX) [file pone.0301869.s002.docx]

**Appendix A – Survey Questionnaire**

| **Survey on Public Awareness of COVID-19 among Seoul Citizens** |
| --- |

※ The survey below is the translated version of the original written in Korean.

| Hello,  Seoul National University's Agricultural and Life Sciences Research Institute is conducting a research project titled "Development of Spatial Models and Guidelines for Infection Disease-Responsive Green Infrastructure for Promoting Mental Health in the Post-COVID-19 Era." This survey is being conducted as part of this project and will serve as important policy research material for preventing and responding to climate change-related damages in the future.  The contents of this survey will be kept strictly confidential in accordance with Articles 33 and 34 of the Statistics Act and will never be used for purposes other than statistical analysis and research objectives. Even though you may be busy, your cooperation in participating in the survey, which will contribute to valuable policy data, is greatly appreciated. Thank you.  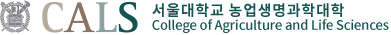  ■ Lead Researcher: Seoul National University College of Agriculture and Life Sciences Research Institute  ■ Survey Administrator: OpenSurvey |
| --- |

| ※ The following is basic information about Coronavirus Disease 2019 (COVID-19). |
| --- |

| ※ Transmission Routes and Government Response System for COVID-19  The primary way COVID-19 spreads is through respiratory droplets, mainly when infected individuals breathe, talk, cough, or sneeze, particularly within a range of about 2 meters.  Research suggests that aside from respiratory droplets, the virus can also spread through surface contact with contaminated objects or through the air. However, airborne transmission typically occurs in specific environments, such as enclosed spaces like medical facilities conducting aerosol-generating procedures, karaoke rooms, coffee shops, and indoor gym facilities, where respiratory droplets linger in the air for extended periods.  Since February 23, 2020, the South Korean government has maintained the crisis alert level for infectious diseases at the "serious" level due to the spread of COVID-19.  To combat the spread of COVID-19, the government has implemented various measures, including promoting social distancing, mandating mask-wearing, requiring the use of entry logs, encouraging vaccination, and enforcing strengthened social distancing measures and vaccination passes based on the severity of COVID-19 prevention measures. |
| --- |

I have confirmed. Yes( )

| ※ The following are questions about Coronavirus Disease 2019 (COVID-19). |
| --- |

1. How much do you know about the COVID-19 you just read?

| ① very little knowledge | ② limited knowledge | ③ moderate knowledge | ④ good knowledge | ⑤ excellent knowledge |
| --- | --- | --- | --- | --- |
| ( ) | ( ) | ( ) | ( ) | ( ) |

2. How much do you trust our government's COVID-19 response system?

| ① strongly distrust | ② distrust | ③ neutral | ④ trust | ⑤ strongly trust |
| --- | --- | --- | --- | --- |
| ( ) | ( ) | ( ) | ( ) | ( ) |

3. Are you afraid of COVID-19?

| ① not at all afraid | ② slightly afraid | ③ moderately afraid | ④ very afraid | ⑤ extremely afraid |
| --- | --- | --- | --- | --- |
| ( ) | ( ) | ( ) | ( ) | ( ) |

4. How often do you check the number of COVID-19 confirmed cases?

① more than 5 times a week ② 3-4 times a week ③ 1-2 times a week ④ 1-3 times a month ⑤ almost never

5~13) How well do you adhere to the following measures for preventing the spread of COVID-19?

| **Actions to prevent the spread of COVID-19** | **not complying at all** | **occasionally not complying** | **average** | **complying most of the time** | **complying very well** |
| --- | --- | --- | --- | --- | --- |
| Avoid unnecessary outings, gatherings, and travel. | ①( ) | ②( ) | ③( ) | ④( ) | ⑤( ) |
| Always wear a mask approved by the Ministry of Food and Drug Safety when going out. | ①( ) | ②( ) | ③( ) | ④( ) | ⑤( ) |
| Wash hands with soap for 30 seconds or more after going out. | ①( ) | ②( ) | ③( ) | ④( ) | ⑤( ) |
| Frequently disinfect and ventilate the surrounding environment. | ①( ) | ②( ) | ③( ) | ④( ) | ⑤( ) |
| Practice cough etiquette (cover mouth and nose with sleeve, tissue, etc.). | ①( ) | ②( ) | ③( ) | ④( ) | ⑤( ) |
| Avoid touching your eyes, nose, and mouth with unwashed hands. | ①( ) | ②( ) | ③( ) | ④( ) | ⑤( ) |
| Practice social distancing in all spaces. | ①( ) | ②( ) | ③( ) | ④( ) | ⑤( ) |
| Avoiding enclosed, crowded, and close-contact spaces. | ①( ) | ②( ) | ③( ) | ④( ) | ⑤( ) |
| Avoid sharing food. | ①( ) | ②( ) | ③( ) | ④( ) | ⑤( ) |

| ※ The following are questions about your activities. |
| --- |

14. What do you prefer more normally (regardless of the COVID-19 pandemic)?

① prefer indoor activities ② prefer outdoor activities

15. How often did you dine out during the COVID-19 pandemic?

① more than 5 times a week ② 3-4 times a week ③ 1-2 times a week

④ 1-3 times a month ⑤ almost never

16. How often did you use café spaces during the COVID-19 pandemic? *Please respond based on dining inside the establishment. (Takeout not included)

① more than 5 times a week ② 3-4 times a week ③ 1-2 times a week

④ 1-3 times a month ⑤ almost never

17. How often did you use indoor complex cultural shopping malls* during the COVID-19 pandemic? *Indoor complex cultural shopping malls: Spaces that include shopping, dining, and some entertainment facilities, similar to COEX, indoor outlets, and Starfield.

① more than 5 times a week ② 3-4 times a week ③ 1-2 times a week

④ 1-3 times a month ⑤ almost never

18. How often did you use parks and open spaces* during the COVID-19 pandemic? *Open spaces refer to parks, green spaces, playgrounds, amusement parks, apartment landscaping areas, and other open community facilities and spaces.

① more than 5 times a week ② 3-4 times a week ③ 1-2 times a week

④ 1-3 times a month ⑤ almost never

19. How much do you think your choice of leisure activity locations has been influenced by COVID-19?

| ① not influenced at all | ② generally not influenced | ③ neutral | ④ influenced to some extent | ⑤ extremely influenced |
| --- | --- | --- | --- | --- |
| ( ) | ( ) | ( ) | ( ) | ( ) |

| ※ From now on, I will ask you questions about your opinion regarding parks and open spaces. Open spaces refer to parks, green spaces, playgrounds, amusement parks, apartment landscaping areas, and other open community facilities and spaces. |
| --- |

20. How important do you think parks and open spaces are?

| ① not important at all | ② not very important | ③ neutral | ④ important | ⑤ very important |
| --- | --- | --- | --- | --- |
| ( ) | ( ) | ( ) | ( ) | ( ) |

21. You referred to the importance of parks and open spaces as 'ANSWERS'. Has your perception of the importance of parks and open spaces changed during the COVID-19 pandemic?

① became more important ② became less important ③ no change

22. If so, did your actual usage of parks and open spaces change at all during the COVID-19 pandemic?

① use more ② use less ③ no change

23. What was the most important aspect of parks and open spaces for you before the COVID-19 pandemic?

① opportunities for environmental education while experiencing nature

② opportunities for physical activity

③ opportunities for stress relief

④ opportunities for educational programs

⑤ provide meeting places

⑥ other (please specify)

24. What is the most important aspect of parks and open spaces for you during the COVID-19 pandemic?

① opportunities for environmental education while experiencing nature

② opportunities for physical activity

③ opportunities for stress relief

④ opportunities for educational programs

⑤ provide meeting places

⑥ other (please specify)

25. What activity do you engage in the most when visiting parks and open spaces?

⓪ usually do not visit parks or open spaces

① walking

② running

③ sitting

④ using personal mobility such as bicycles, kick scooters, inline skates, etc.

⑤ other (please specify)

26. Are there easily accessible parks and open spaces in your daily life?

① yes ② no

27. Where is the park or open space closest to you that you can easily access on foot in your daily life?

*Please respond based on parks and open spaces you are familiar with for easy access, regardless of whether you actually use them.

⓪ nowhere easily accessible on foot ① near home ② near workplace ③ other (please specify)

28. How often do you use parks and open spaces that are easily accessible on foot in your daily life?

| ① never use | ② rarely use | ③ occasionally use | ④ frequently use | ⑤ very frequently use |
| --- | --- | --- | --- | --- |
| ( ) | ( ) | ( ) | ( ) | ( ) |

29. What is the main reason for you facing constraints in engaging in outdoor activities?

⓪ no constraints on outdoor activities ① lack of time ② lack of space

③ fear of covid-19 ④ other (please specify)

30. Do you think activities in parks and open spaces are safe from COVID-19 infection?

| ① not at all true | ② mostly not true | ③ neutral | ④ mostly true | ⑤ very true |
| --- | --- | --- | --- | --- |
| ( ) | ( ) | ( ) | ( ) | ( ) |

31. What do you consider the most important factor in parks and open spaces?

⓪ there are no particularly important factors.

① walking trails and hiking paths

② benches

③ grass fields

④ plazas

⑤ exercise equipment

⑥ water features (fountains, lakes, etc.)

⑦ trees and flowers

⑧ other (please specify)

32. Do you think the following spaces are essential for modern people experiencing the COVID-19 pandemic? Parks and open spaces.

① yes ② no

33. Where did you most frequently visit parks and open spaces before the COVID-19 pandemic?

⓪ none in particular / do not use parks or open spaces

① large theme parks (sports parks, ecological parks, children's parks, etc.)

② apartment complex landscaping areas

③ small neighborhood parks near home

④ small neighborhood parks near work

⑤ landscaping areas within the workplace

⑥ linear parks focused on walking

⑦ waterfront parks (lake parks, riverbanks)

⑧ other (please specify)

34. Where have you most frequently visited parks and open spaces during the COVID-19 pandemic?

⓪ none in particular / do not use parks or open spaces

① large theme parks (sports parks, ecological parks, children's parks, etc.)

② apartment complex landscaping areas

③ small neighborhood parks near home

④ small neighborhood parks near work

⑤ landscaping areas within the workplace

⑥ linear parks focused on walking

⑦ waterfront parks (lake parks, riverbanks)

⑧ other (please specify)

35. How much time are you willing to spend traveling to parks and open spaces? *Please respond with the time you are willing to spend traveling, regardless of the mode of transportation.

① less than 1 minute to 5 minutes

② less than 5 minutes to 10 minutes

③ less than 10 minutes to 30 minutes

④ less than 30 minutes to 1 hour

⑤ more than 1 hour

36. To what extent do you agree with the following statement? I occasionally take off my mask when going to parks and open spaces.

| ① not at all true | ② mostly not true | ③ neutral | ④ mostly true | ⑤ very true |
| --- | --- | --- | --- | --- |
| ( ) | ( ) | ( ) | ( ) | ( ) |

37. To what extent do you agree with the following statement? I feel anxious about COVID-19 when going to parks and open spaces.

| ① not at all true | ② mostly not true | ③ neutral | ④ mostly true | ⑤ very true |
| --- | --- | --- | --- | --- |
| ( ) | ( ) | ( ) | ( ) | ( ) |

38. What is your biggest concern when going to parks and open spaces during the COVID-19 pandemic? *Please respond, even if you did not visit parks and open spaces during the pandemic.

⓪ no particular concerns

① worried that it may be impossible to practice social distancing in parks and open spaces

② worried about not being able to maintain social distancing while moving around

③ worried about frequent changes in the park's rules for coping with the covid-19 pandemic

④ worried that others may take off their masks

⑤ worried about inadequate restroom facilities

⑥ other (please specify)

39. Do you think parks and open spaces are more necessary now than before in the following situation? The post-COVID era.

| ① not at all true | ② mostly not true | ③ neutral | ④ mostly true | ⑤ very true |
| --- | --- | --- | --- | --- |
| ( ) | ( ) | ( ) | ( ) | ( ) |

40. What do you think is the most important type of parks and open spaces in the post-COVID era, after the end of the pandemic?

⓪ no particular significant type

① linear parks and tree-lined streets for park-like walking paths

② ecological parks for learning about nature

③ parks where indoor activities such as fitness, arts, culture, and social gatherings can be moved outdoors

④ parks near home regardless of size

⑤ parks within or near the workplace

⑥ other (please specify)

41. Did using parks and open spaces during the COVID-19 pandemic help relieve stress?

| ① not at all true | ② mostly not true | ③ neutral | ④ mostly true | ⑤ very true |
| --- | --- | --- | --- | --- |
| ( ) | ( ) | ( ) | ( ) | ( ) |

42. Did you feel the need to visit parks and open spaces more often after using them during the COVID-19 pandemic?

| ① not at all true | ② mostly not true | ③ neutral | ④ mostly true | ⑤ very true |
| --- | --- | --- | --- | --- |
| ( ) | ( ) | ( ) | ( ) | ( ) |

43. Did you feel physically healthier after using parks and open spaces during the COVID-19 pandemic?

| ① not at all true | ② mostly not true | ③ neutral | ④ mostly true | ⑤ very true |
| --- | --- | --- | --- | --- |
| ( ) | ( ) | ( ) | ( ) | ( ) |

| ※ Now, I will ask demographic questions for statistical classification. |
| --- |

44. What do you think about your health usually?

| ① very bad | ② bad | ③ neutral | ④ good | ⑤ very good |
| --- | --- | --- | --- | --- |
| ( ) | ( ) | ( ) | ( ) | ( ) |

45. How often do you engage in vigorous physical activities (such as jogging, working out, swimming) that make you very tired or breathless? * Please respond based on activities lasting more than 20 minutes.

① more than 5 times a week ② 3-4 times a week ③ 1-2 times a week

④ 1-3 times a month ⑤ almost never

46.How often did you walk for at least 30 minutes once a week in the past week?

① more than 5 times a week ② 3-4 times a week ③ 1-2 times a week

④ 1-3 times a month ⑤ almost never

47.Have you ever experienced "corona blues" (depression caused by the COVID-19 pandemic) during the pandemic period?

① yes ② no

48 to 61: Please fill in the blanks and mark with a circle (○) or a checkmark (√) where appropriate.

| 48. Gender | ① male ② female | 49. Age |  |
| --- | --- | --- | --- |
| 50. Number of Household Members | ① one (living alone)  ② two  ③ three  ④ four  ⑤ five or more | 51. Marital status | ① married  ② single  ③ other (divorced, separated, etc.) |
| 52. Number of Children | ① no children  ② one  ③ two  ④ three  ⑤ four  ⑥ five or more | 53. COVID-19 infection status | 1. experienced infection 2. no experience of infection |
| 54. The COVID-19 infection status of family and acquaintances | ① family/friends experienced infection  ② no family/friends experienced infection | 55. Primary mode of transportation | ① walking  ② public transportation  ③ personal vehicle  ④ bicycle, kick scooter, etc.  ⑤ other (please specify) |
| 56. Vaccination status | ① not vaccinated  ② received johnson & johnson first dose  ③ received astrazeneca/moderna/pfizer first dose  ④ received astrazeneca/moderna/pfizer second dose  ⑤ received booster shot | | |
| 57. Housing occupancy type | ① ownership  ② lease  ③ monthly rent  ④ deposit lease (lease + monthly rent)  ⑤ other ( ) | | |
| 58. Type of housing | ① apartment  ② residential facility within a commercial building ③ house (only one household resides in a house) ④ studio apartment  ⑤ share house ⑥ dormitory ⑦ other (please specify) | | |
| 59. Household income (monthly) | ① less than 1 million won ② 1 million to less than 2 million won ③ 2 million to less than 3 million won ④ 3 million to less than 5 million won ⑤ 5 million to less than 7 million won ⑥ 7 million won or more | | |
| 60. Education | ① elementary school graduate or less  ② high school graduate  ③ college graduate  ④ graduate school graduate | | |
| 61. Occupation | ① college student/graduate student ② indoor office work - office workers and professionals ③ indoor non-office work (self-employed in stores, shop assistants, indoor production workers, housekeepers, etc.) ④ outdoor non-office work (construction sites, welding, security guards, outdoor production workers, delivery, agriculture, etc.) ⑤ homemaker ⑥ unemployed/retired ⑦ other (please specify) | | |

62. How much do you agree with the following statement? Concerns about COVID-19 infection in the workplace (or school)

| ① not at all agree | ② mostly not agree | ③ neutral | ④ mostly agree | ⑤ very agree |
| --- | --- | --- | --- | --- |
| ( ) | ( ) | ( ) | ( ) | ( ) |

63. How well do you adhere to the following infection control measures for preventing the spread of COVID-19? Always wearing KFDA-approved masks indoors (at work/school, etc.)

| ① not complying  at all | ② occasionally not complying | 1. average | 1. complying   most of the time | 1. complying very well |
| --- | --- | --- | --- | --- |
| ( ) | ( ) | ( ) | ( ) | ( ) |

64. Have you experienced remote work during the COVID-19 pandemic?

① Yes

② No

65. Please provide the district and neighborhood information of your current residence. Example: Seoul, Gwangjin-gu, Hwayang-dong.

| **Residence** | Seoul, ( )Gu ( )Dong |
| --- | --- |

Thank you very much for taking the time to complete the survey.
